# Current situation and future direction of Newcastle disease vaccines

**DOI:** 10.1186/s13567-022-01118-w

**Published:** 2022-11-26

**Authors:** Zenglei Hu, Xiaozheng He, Jing Deng, Jiao Hu, Xiufan Liu

**Affiliations:** 1 Joint International Research Laboratory of Agriculture and Agri-Product Safety, Ministry of Education of China, Yangzhou University, Yangzhou, China; 2 Animal Infectious Disease Laboratory, School of Veterinary Medicine, Yangzhou University, Yangzhou, China; 3 Jiangsu Key Laboratory of Zoonosis, Yangzhou University, Yangzhou, China; 4 Jiangsu Coinnovation Center for Prevention and Control of Important Animal Infectious Diseases and Zoonoses, Yangzhou University, Yangzhou, China

**Keywords:** Newcastle disease virus, vaccines, vaccine profiles, vaccine development

## Abstract

Newcastle disease (ND) is one of the most economically devastating infectious diseases affecting the poultry industry. Virulent Newcastle disease virus (NDV) can cause high mortality and severe tissue lesions in the respiratory, gastrointestinal, neurological, reproductive and immune systems of poultry. Tremendous progress has been made in preventing morbidity and mortality caused by ND based on strict biosecurity and wide vaccine application. In recent decades, the continual evolution of NDV has resulted in a total of twenty genotypes, and genetic variation may be associated with disease outbreaks in vaccinated chickens. In some countries, the administration of genotype-matched novel vaccines in poultry successfully suppresses the circulation of virulent NDV strains in the field. However, virulent NDV is still endemic in many regions of the world, especially in low- and middle-income countries, impacting the livelihood of millions of people dependent on poultry for food. In ND-endemic countries, although vaccination is implemented for disease control, the lack of genotype-matched vaccines that can reduce virus infection and transmission as well as the inadequate administration of vaccines in the field undermines the effectiveness of vaccination. Dissection of the profiles of existing ND vaccines is fundamental for establishing proper vaccination regimes and developing next-generation vaccines. Therefore, in this article, we provide a broad review of commercial and experimental ND vaccines and promising new platforms for the development of next-generation vaccines.

## Introduction

### Overview of Newcastle disease

Newcastle disease (ND) is an important poultry infectious disease with a history of nearly a century that has caused at least four panzootics globally [1]. ND is caused by virulent Newcastle disease virus (NDV) strains. The first panzootic, from the 1930s to 1960s, was caused by viruses of genotypes I, II, III and IV. The second panzootic, from the late 1960s to 1973, was mainly caused by genotype V and VI viruses. In 1975, the third panzootic started in pigeons and spread to various regions around the world. Genotype VI NDV was responsible for this panzootic. From the late 1980s, genotype VII NDV originated from the Far East and spread throughout the world, causing the fourth panzootic [2]. Currently, genotype VII NDV is endemic in many countries in Asia and Africa, posing a great threat to the poultry industry [3–6].

**Figure 1:**
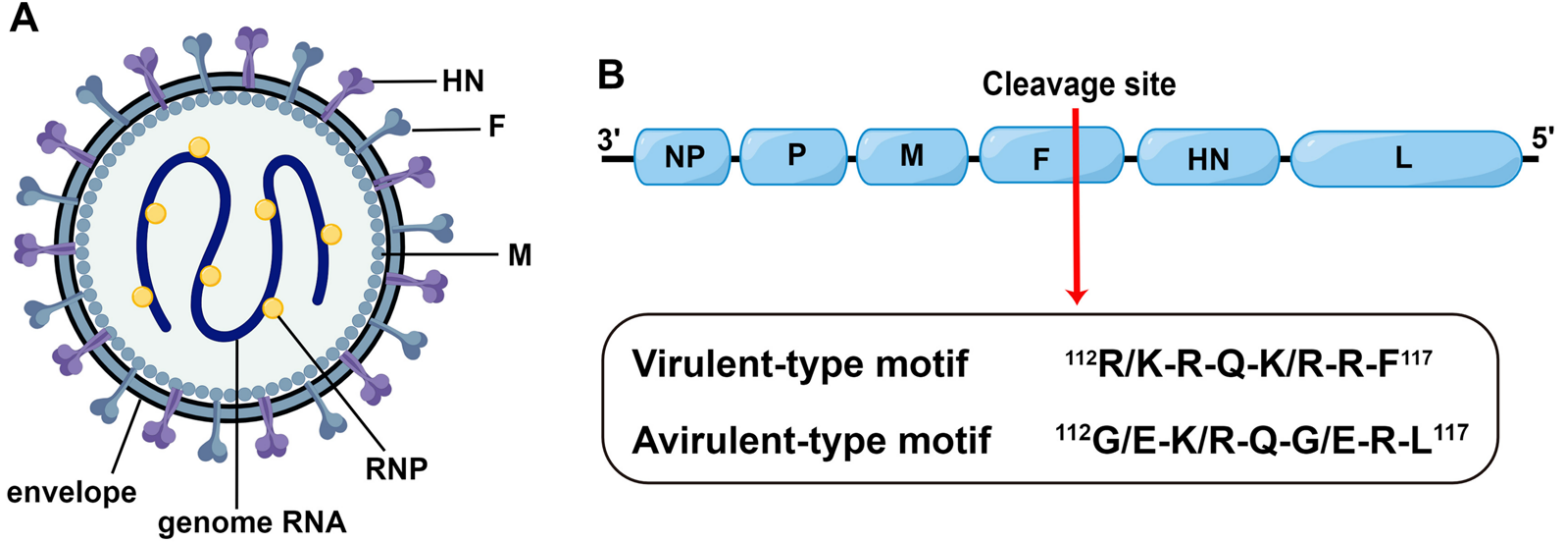
**Schematic illustration of NDV particle and genome structure. A** Illustration of the structure of NDV particles. HN, haemagglutinin-neuraminidase; F, fusion protein; M, matrix protein; RNP, ribonucleotide-protein complex. **B** Schematic representation of the structure of the NDV genome. The red arrow indicates the cleavage site of the F protein. The representative amino acid motifs of the virulent- and avirulent-type cleavage sites are shown.

NDV belongs to the genus *Orthoavulavirus* of the subfamily *Avulavirinae* [7]. NDV is an enveloped virus with a single-stranded, negative-sense, nonsegmented RNA genome (Figure 1A). The NDV genome encodes six viral proteins: the nucleoprotein (NP), phosphoprotein (P), matrix (M), fusion (F), haemagglutinin-neuraminidase (HN) and large (L) proteins [8] (Figure 1B). Based on the pathogenicity observed in susceptible chickens, NDV can be classified into three pathotypes: velogenic, mesogenic and lentogenic [9]. Strains with an intracerebral pathogenicity index (ICPI) ≥ 0.7 are defined as virulent strains according to the standards of the World Organisation for Animal Health (OIE) [9]. Virulent strains cause systematic infection and severe lesions in chickens [10], and the amino acid at the cleavage site of the F protein is the major determinant of virulence [11].

### Evolution of NDV

Nearly 100 years after its emergence, NDV has undergone remarkable evolution, resulting in high diversity in terms of genetics, virulence, antigenicity and host range. First, similar to other nonsegmented RNA viruses, genomic changes in NDV mainly stem from the error-prone nature of the polymerase, which generates genetic variants known as quasispecies. Virus quasispecies harbouring site mutations accumulating in the NDV genome can lead to apparent changes in viral phenotypes under selection pressure, representing a primary mechanism of virus evolution [12–14]. Novel genotypes may emerge with the accumulation of genetic variations, which may explain the association between each ND panzootic and the emergence of new genotypes. In addition to natural genetic evolution, antibodies in poultry flocks immunized with NDV vaccines exert high immune pressure on virus survival [15], especially in countries where extensive and frequent ND vaccination is performed. Studies have verified that mutations in the two surface glycoproteins, F and HN, contribute to viral escape from antibody immunity [16, 17]. Second, the change in the host range is also an important outcome of virus evolution. Chickens are the major host of NDV, but there is substantial expansion of the host range after ND panzootics. Waterfowl are the natural hosts of lentogenic NDV and are thought to be resistant to virulent NDV. However, during the fourth panzootic, outbreaks in geese caused by genotype VII NDV occurred in China, demonstrating the spillover of NDV from terrestrial to aquatic birds [3, 18]. Subsequently, ND outbreaks in ducks were reported more frequently than before, highlighting an increased threat of NDV to waterfowl [19–21]. Another unique feature of the NDV host range is virus circulation in pigeons. Since the third panzootic, the primary genotype spreading in pigeons has been genotype VIb, and this genotype mainly infects pigeons under natural conditions [22–24]. This finding indicates that genotype VIb viruses have established steady host specificity in pigeons since the third panzootic.

In addition, antigenic variation, another important manifestation of virus evolution, may result in immune escape and insufficient protection against dominant viruses. Currently, the most widely used ND vaccines belong to early genotypes, such as genotypes I and II, which were isolated approximately 70 years ago. Nevertheless, the prevalent NDV strains in poultry belong to late genotypes, including genotype V in America, genotype VII in Asia and Africa and genotype VI in pigeons on different continents, which are genetically and antigenically distinct from traditional vaccines. Many studies have consistently verified that conventional ND vaccines protect against morbidity and mortality, rather than reducing virus shedding from vaccinated chickens [25–29]. In these circumstances, field viruses can still be silently disseminated in poultry flocks and cause nontypical diseases.

Due to the low similarity (approximately 87–89%) of the major protective antigens between current vaccines and prevalent strains, the comparison of the merits and deficiencies of commercial and experimental ND vaccines is of paramount importance to formulate effective vaccination programmes and clarify the route for the development of next-generation ND vaccines.

## Commercial ND vaccines

Vaccination against NDV needs to achieve three main goals: alleviation of morbidity and mortality; reduction of virus spreading and shedding in the flock; and protection against virus infection. However, due to the limited practicability of effective tools and skills for assessing virus shedding in the field, the majority of current vaccines and vaccination regimes primarily aim to mitigate clinical disease. In addition, ND vaccines provided by most companies are only required to prevent 90% of morbidity and mortality, while no requirements for reducing virus shedding are stipulated. Notably, the reduction of virus shedding is included as a key parameter for evaluating the efficacy of a novel recombinant genotype VII NDV vaccine in China (see Sect. 2.2). Moreover, vaccination is the last and not the first line of defence against animal infectious diseases, and strict biosecurity measures can lower the risk of animals being exposed to viruses. Vaccination efficiency is also affected by nutritional deficiency, stress, and immunosuppression caused by coinfection with immunosuppressive pathogens such as infectious bursa disease virus (IBDV). Therefore, to achieve eligible herd immunity induced by any ND vaccination strategy, a sufficient vaccine dose must be delivered to at least 85% of animals, and a haemagglutination-inhibition (HI) antibody titre ≥ 3 log_2_ must be triggered in these animals [30]. ND vaccination regimes should be tailored according to the specific conditions of the farm. Thus, the selection of appropriate vaccines is critical for the effectiveness of vaccination and disease control.

### Live vaccines

Live ND vaccines include lentogenic and mesogenic vaccines. Globally, lentogenic live vaccines are the most widely used, and La Sota and B1 are the representative strains. These viruses belong to genotype II and show high similarities at the genetic and antigenic levels. Lentogenic vaccines can induce protective antibody responses, although they differ in tissue tropism and replication pattern in chickens [31]. The La Sota strain shows high tropism to the respiratory system and replicates to high levels in chickens [32]. Antibody titres induced by La Sota are generally high, and this vaccine is thus suitable for use in countries where virulent NDV is endemic. The VG/GA vaccine is characterized by a dual tropism to the respiratory tract and intestine, with a higher tropism to the latter, and can elicit strong mucosal immunity [32]. In addition, the B1 vaccine has a very low virulence and is highly safe to chicks [33], and it is usually used under low-level infection or in chicks. Another type of lentogenic live vaccine is based on genotype I, and V4 and I-2 are representatives of this type. These strains present low virulence and good safety in chickens of all ages [33, 34]. Moreover, V4 and I-2 are typical thermostable vaccines with the unique advantages of being used in remote country areas with limited cold chain facilities, which can be administered through drinking water and food intake [35–37].

Live ND vaccines present good safety because the vaccine strains present low or no virulence and usually cause few vaccinal reactions in chickens. In addition, live vaccines can induce mucosal, humoral and cellular immunity and can be administered through spraying or drinking water. However, under certain conditions, live vaccines may cause undesirable vaccinal reactions, such as growth retardation, mild respiratory signs, and even mortality and increased susceptibility to other pathogens [38]. A cold chain facility is required for the transportation and handling of these vaccines. In addition, live vaccines are generally applied in young chicks, and the presence of maternally derived antibodies (MDAs) may interfere with their efficacy [39, 40]. Therefore, the administration of live ND vaccines at the proper time point based on MDA monitoring is essential for vaccine efficacy.

Another type of live vaccine is based on mesogenic strains such as Mukteswar (genotype III), Komarov (genotype II) and Roakin (genotype II) [41]. These strains show high virulence and can cause mortality in young chickens [42]. Mesogenic live vaccines are highly immunogenic and can provoke a fast and long-lasting antibody response; these vaccines can be used in adult chickens for booster or emergency vaccination. However, because mesogenic viruses are defined as virulent by OIE and increased virulence of this vaccine type has been reported [43], mesogenic live vaccines are largely banned in many countries [41].

Live ND vaccines are produced in specific-pathogen-free (SPF) embryonated chicken eggs (ECEs). The vaccine master seed is inoculated into the allantoic cavity of 9- to 11-day-old ECEs, and the allantoic fluids are collected after incubation [9]. After passing a series of quality control tests, the allantoic fluids supplemented with stabilizers are subjected to lyophilization [9, 41]. This process is critical for the maintenance of the antigen content and shelf life of vaccines. In addition, the determination of the 50% embryo infectious dose (EID_50_) is a key checkpoint for assessing the antigen level and efficacy of live vaccines. Studies have validated the correlation between the vaccine dose (in EID_50_) and protection efficacy [44]. Under experimental conditions, SPF chickens vaccinated with 10^4^–10^5^ EID_50_ of live La Sota vaccine are protected against morbidity and mortality, although the infection and replication of the challenge virus are not reduced [44]. A dose of 10^6^ EID_50_ is the minimum requirement for live vaccines to provide full protection and decrease virus shedding [44]. Therefore, it is argued that three goals of ND vaccination can be achieved when the vaccine dose is high enough. However, in practice, an increase in the vaccine dose leads to increased costs of disease control, which makes their use in the poultry industry unfeasible.

A major concern related to current live vaccines is their mismatch with the dominant viruses. Although NDV belongs to a single serotype, there are great genetic and antigenic variations between conventional live vaccines and viruses present in the field. Many studies have revealed that the amino acid sequence homologies of the F and HN proteins between the La Sota strain and genotype VII strains range from 87–89% and 87–88%, respectively [28, 45]. Similarly, in Latin America, the most frequent genotypes affecting poultry farms correspond to genotypes V, VI, VII, XII and XVI [46], and the identities between La Sota and the prevalent strains are 87–89% [47]. There are two conflicting perspectives regarding this issue. Some reports have pointed out that the antigenic difference between the vaccines and prevalent strains is not the leading cause of disease outbreaks in the field. Instead, poor flock immunity, caused by inadequate vaccination practices, may be responsible for the low protection provided by live vaccines [48–50]. These findings highlight the importance of sufficient vaccination for the efficacy of live vaccines in the field. On the other hand, the administration of live ND vaccines homologous to circulating viruses is beneficial for reducing virus shedding in the flock. One experimental and two commercial live recombinant genotype VII vaccines provided better protection against field isolates in chickens than La Sota [28, 51]. Accordingly, a live recombinant NDV expressing the F and HN genes from genotype V NDV was more effective in reducing viral excretion than the La Sota strain [46]. Moreover, solid evidence supporting this conclusion was obtained from studies on inactivated ND vaccines, as discussed in detail below. Mechanistically, a titre-dependent escape model was recently proposed, and in this model, the vaccine virus can be neutralized by low antibody titres induced by the homologous vaccine, whereas higher antibody titres are needed to neutralize the field strains harbouring variations in the major neutralizing epitopes. This model supports the requirement to develop vaccines homologous to field viruses [52]. Therefore, both genotype-matched vaccines and proper vaccination operations are essential to achieve good protection in the field.

The primary challenge for generating genotype-matched live vaccines against virulent NDV is how to confer antigenicity as well as safety. Two main strategies are employed to generate genotype-matched live vaccines. Some virulent strains have been attenuated using reverse genetics and engineered to obtain modified live vaccines [28, 53, 54]. Virulent strains are attenuated by modifying the F cleavage site, and extra specific mutations in the L protein can further guarantee the safety of the attenuated virus [54]. No live attenuated vaccines generated using this strategy have yet been commercialized. The other strategy is to replace the protective F and HN antigens of a lentogenic virus with the corresponding proteins from prevalent virulent strains, and the F cleavage site is usually mutated [55]. Several live recombinant NDV vaccines of this type have been commercialized in Korea (Himmvac Dalguban N (Plus) Live Vaccine), Egypt (live attenuated RINNOVAC™ELI-7) and Mexico (Genovax N5). Additionally, the origin of lentogenic NDV backbones may impact the efficacy of the recombinant vaccines [51]. Notably, the safety of these virulent virus-derived live vaccines should be carefully monitored, even when studies under well-controlled conditions in the laboratory verified safety in experimental animals.

### Inactivated vaccines

Inactivated vaccines are also extensively used for disease control. Allantoic fluids are harvested from ECEs inoculated with the master seed and are inactivated with formalin or β-propiolactone [9, 41]. The inactivation of allantoic fluids must be performed multiple times to ensure the complete loss of infectivity. Adjuvants such as mineral oil are added to inactivated allantoic fluids to prepare emulsified vaccines. The cost of the production of inactivated vaccines is relatively high. Lentogenic NDV strains, including La Sota, Ulster and B1, are usually used as the master seed due to their high virus yield in ECEs. The safety of inactivated vaccines is good because the viruses cannot replicate and spread among vaccinated chickens. Inactivated vaccines are administered individually via a parenteral route such as intramuscular or subcutaneous (s.c.) injection, making the process time consuming and labour intensive. These vaccines mainly induce high, long-lasting humoral immunity, whereas they are poor inducers of cellular or mucosal immune responses.

Similar to live vaccines, the efficacy of inactivated vaccines is affected by the degree of matching with circulating viruses. In most countries where ND is endemic, inactivated vaccines based on the La Sota strain are administered in poultry. Notably, the amino acid identities of the F and HN proteins between the traditional vaccine strains and the dominant NDVs are 87–89% and 87–88%, respectively [26, 48]. La Sota-based inactivated vaccines elicit significantly lower HI titres against a heterologous virus than against a homologous antigen [27, 47]. Moreover, chickens vaccinated with conventional vaccines are completely protected from morbidity and mortality, although they can still shed large amounts of virus [25, 26, 29]. These findings may explain the phenomenon of the frequent occurrence of nontypical ND in flocks vaccinated with traditional vaccines.

In contrast to the situation in live attenuated vaccines, it is easier to solve the problem of genotype matching in inactivated ND vaccines using reverse genetics. Recombinant vaccines specific to genotype VII NDV have been generated via different strategies [27, 28, 55, 56]. Some of these vaccine candidates have already been commercialized in different countries [27, 57]. A Korean team developed a recombinant genotype VII vaccine candidate (KBNP-C4152R2L) by expressing the F and HN genes in the La Sota backbone [55], and this candidate has been commercialized in the form of live and inactivated vaccines. Studies verified that this vaccine can confer better protection than La Sota in terms of reducing virus shedding [51, 57]. In addition, our team developed an attenuated genotype VII NDV vaccine (A-VII) by mutating the amino acid motif in the F cleavage site [27]. Compared to the La Sota vaccine, the inactivated A-VII vaccine induces a faster and stronger antibody response, fully protecting chickens from challenge with genotype VII virus and significantly decreasing virus shedding. Notably, the reduction of virus shedding was included for the first time in the Chinese Veterinary Pharmacopoeia as a key standard for the efficacy testing of ND vaccines. Cloacal swabs of at least 7 out of 10 vaccinated chickens should be negative for virus isolation on Day 5 post challenge [58]. The A-VII vaccine can also be used for disease control in geese [27, 58]. After the commercialization of A-VII in 2014 and its extensive application in poultry thereafter, the incidence of ND decreased dramatically, and the circulation of virulent genotype VII NDV in poultry flocks was largely abolished in China (based on data from the China Official Veterinary Bulletin). Genotype-matched vaccines are homologous to prevalent viruses and are more effective in preventing virus shedding. Therefore, a question arises regarding whether genetic or antigenic variations are more likely to occur in field viruses under immune pressure from homologous vaccines. Using an in vitro serum-neutralizing assay, a recent study highlighted that there were no significant differences in the variation of the F and HN genes of genotype VII NDV under selection pressure from homologous and heterologous vaccines [59]. This finding indicates that the application of genotype-matched vaccines may not change the variation of genotype VII NDV.

In addition to vaccines for chickens and geese, there is a great demand for ND vaccines specialized for pigeons. Distinct from NDVs of other host origins, pigeon-originating viruses, also known as pigeon paramyxovirus (PPMV)-1 (mainly genotype VIb) exhibit host specificity to pigeons. This genotype is also a late genotype, showing high genetic and antigenic variation relative to traditional vaccine strains [60]. Inactivated vaccines based on the La Sota or Ulster strain can provide complete protection against PPMV-1 infection, whereas they induce lower HI titres against PPMV-1 and fail to reduce virus shedding in pigeons [61]. Only inactivated vaccines are available for pigeons, such as AVIPRO 111 PMV1 and Nobilis Paramyxo P201 (data from the PoultryMed). Moreover, PPMV-1 can be engineered by reverse genetics to increase its safety, antigenicity and virus yield, which may represent a future direction for the development of pigeon ND vaccines.

### Viral-vectored vaccines

#### Fowlpox virus-vectored vaccines

The generation of multivalent vaccines can increase the spectrum of vaccines and efficiency of vaccination in the poultry industry. Traditionally, the production of multivalent poultry vaccines requires a certain antigen concentration to maintain a sufficient dose of each antigen, which undoubtedly complicates the entire process and increases the cost. Alternatively, virus vector platforms have emerged as promising systems for the development of bivalent or multivalent poultry vaccines [62]. Protective antigen genes of target pathogens are inserted into the genome of the vector virus using molecular biological technology, and the recombinant viruses express foreign antigens in the process of virus replication. Compared to traditional multivalent vaccines, there are several advantages of virus-vectored vaccines, including the coexpression of multiple protective antigens in a single virus vector, the lack of a need for antigen concentration, the induction of humoral, cellular or mucosal immunity, high genetic stability and safety.

Since the 1990s, scientists have expressed the F or HN genes of NDV in the genome of fowlpox virus (FPV), and the resulting recombinant vaccines can protect chickens against challenge with virulent NDVs [63–66]. At least two commercial FPV-vectored ND vaccines are available on the market, including TROVAC^®^-NDV from Boehringer Ingelheim and VECTORMUNE^®^ FP-N from Ceva. FPV is the largest animal virus, and its genome has a high capacity for including foreign genes. In addition, FPV has a restricted host range and therefore shows good safety among other animal species. FPV is commonly propagated in SPF ECEs or chicken embryo fibroblasts, and chorioallantoic membrane or cell cultures are collected for lyophilization with stabilizer supplementation. Immunization with FPV-vectored ND vaccines is performed through s.c. injection or wing-web stabbing, and their mass administration is therefore not possible. In addition, the presence of high MDA titres against FPV in commercial chickens strongly impairs the efficacy of PFV-vectored vaccines [67]. These disadvantages of the FPV vector mean that its use in ND vaccine delivery is highly limited.

#### Vaccines based on turkey herpesvirus

ND vaccines based on turkey herpesvirus (HVT) are a successful example of commercial virus-vectored poultry vaccines. HVT, also known as serotype 3 Marek’s disease virus (MDV), is one of the most widely used viruses for generating novel vector vaccines. In the early 1990s, the F gene of NDV was inserted into the genome of HVT, and the resultant recombinant virus can provide dual protection against NDV and MDV [68]. There are four commercial HVT-vectored ND vaccines available on the market, including Vectormune® ND from Ceva, Innovax-ND from MSD, Poulvac Procerta HVT-ND from Zoetis and NEWXXITEK™ HVT + ND from Boehringer Ingelheim (data from the PoultryMed). The Vectormune® ND vaccine elicits delayed but durable antibody immunity and provides good protection against NDV challenge [69]. The F gene of the genotype I NDV D26-76 strain is expressed in the HVT FC126 strain. Although the F gene donor virus is distinct from circulating viruses, the Vectormune^®^ ND vaccine can still provide protection against heterologous genotypes (IV, V and VII) and reduce virus shedding [70–72]. In particular, the HVT-vectored ND vaccine decreases virus shedding more efficiently when genotype VII NDV challenge is conducted via the intramuscular route compared to the intranasal route, suggesting that the vaccine induces strong systematic immunity [70]. In low-ND-risk regions, the administration of the Vectormune^®^ ND vaccine through the in ovo injection of 18-day embryos or s.c. injection in day-old chicks can confer sufficient protection. In intermediate- and high-ND-risk regions, it is recommended that early prime immunization is performed using the Vectormune^®^ ND vaccine and booster immunization with live vaccines after 2–3 weeks to establish solid protection. The merits of the HVT vector include the following: (1) the HVT genome is large, with a high capacity for including multiple foreign genes; (2) MDA interference with HVT is low, allowing early vaccination in chickens; (3) the mass administration of HVT-vectored vaccines can be performed in hatchery via in ovo injection or s.c. injection in day-old chicks; (4) HVT-vectored vaccines can induce life-long protection due to the persistent infection of HVT; (5) HVT-vectored vaccines can induce both antibody and cellular immunity. These strengths underlie the success of HVT-vectored vaccines worldwide. In addition, the CRISPR/Cas9 genome editing tool can be used as a simple and rapid approach for developing recombinant HVT-vectored vaccines [73]. However, the HVT vector is characterized by some limitations, such as delayed onset of immunity and the inability to administer a second HVT vaccine in the same flock [1, 62]. Moreover, because HVT vaccines are presented in freeze-dried or frozen cell suspension formulations, a cold chain or liquid nitrogen is required for the storage of vaccines, which may restrict their application in the areas lacking required facilities.

#### NDV-vectored vaccines

NDV is a good vector for generating bivalent or multivalent vaccines against ND and other infectious poultry diseases [74, 75]. There are some strengths of NDV when used as a vaccine vector: (1) the genome of NDV is ~15 kb, allowing easy molecular manipulation; (2) NDV presents a high virus yield in ECEs; (3) NDV can accommodate and express a foreign gene stably; (4) NDV shows a low risk of gene exchange and recombination; (5) NDV can induce mucosal, humoral and cellular immunity; and (6) NDV vaccines can be administered by mass vaccination approaches. Numerous vaccine candidates based on NDV vectors have been generated, including candidates targeting avian influenza virus (AIV), IBDV and infectious bronchitis virus (IBV) [74, 75]. Two H5 subtype AIV vaccines based on NDV vectors have been commercialized in China and Mexico [76, 77].

The majority of bivalent vaccines are based on lentogenic NDVs, and the noncoding region between the P and M genes is the optimal insertion site for foreign genes [78]. The common strategy of vaccine construction is to insert foreign genes as an independent transcription unit in the NDV genome, and transcriptional signals, including the gene end, intergenic and gene start sequences, are added upstream of the foreign genes. Recombinant NDV can be rescued using reverse genetics, and virus replication, foreign protein expression, virulence, genetic stability and efficacy in animals are then systematically assessed.

Although NDV-vectored vaccines are promising, interference from MDA is a major concern related to their clinical application [74]. Using the common strategy of vaccine generation, foreign antigens are incorporated into the envelope of virus particles [79]. Therefore, antibodies against NDV as well as foreign antigens can impact vectored vaccines. Supporting evidence was obtained from a previous study demonstrating stronger interference from the anti-H5 antibody with the NDV-vectored H5 vaccines than with antibodies against the vector in chickens [80]. Researchers have made many efforts to solve the problem of MDA interference and have adopted two main strategies, “antigen camouflage” and “antigen decoy”. First, regarding interference with the vector, the “antigen camouflage” strategy was developed based on poor cross-reactivity between NDV and other avian paramyxovirus (APMV) serotypes [81]. The F and HN genes of NDV are replaced by the corresponding genes from APMV-2 or -8, and the resultant chimeric vector is not influenced by pre-existing NDV antibodies in chickens. Recombinant vaccines based on chimeric vectors are highly immunogenic and efficacious in MDA-positive chickens [82–84]. Second, the “antigen decoy” strategy for antagonizing interference from foreign antigens was recently reported. Using the H5 subtype AIV as a model, haemagglutinin (HA) has been expressed in the full-length and secreted versions in the NDV vector [85]. The secreted form acts as a decoy that can absorb a fraction of the pre-existing HA-specific antibodies. The membrane-anchored HA protein is thus protected from binding by HA antibodies, allowing these antigens to elicit specific immunity. Therefore, we recent proposed that the combination of the “antigen camouflage” and “antigen decoy” strategies may provide a viable pathway for developing novel MDA-resistant NDV-vectored vaccines [86].

## Experimental ND vaccines

### Recombinant subunit vaccines

Currently, almost all ND vaccines are produced in ECEs, which are a traditional, mature system for poultry vaccine production. It is easy to perform large-scale ECE culture, and NDV has a high virus yield in ECEs. However, there are still some shortcomings of the egg-based system, including high costs, unstable supply (especially during disease outbreaks), production of a large amount of biowaste, high energy consumption and carbon dioxide emissions from biowaste treatment. Therefore, it is necessary to develop alternative platforms for poultry vaccine production.

The baculovirus expression vector system (BEVS) is a promising alternative to the egg-based system. Several commercial human and animal vaccines are produced via the BEVS [87–90]. A recombinant baculovirus expressing foreign antigens is generated and propagated in insect cells for large-scale production. There are some prominent advantages of the BEVS: (1) the posttranslational modification function of insect cells is suitable for the expression of viral glycoproteins; (2) large-scale fermentation culture of insect cells in serum-free medium achieves a high antigen yield with low costs; (3) the baculovirus shows high host specificity and is safe for vertebrate animals; (4) the baculovirus genome has a large capacity for coexpressing multiple foreign antigens; and (5) the baculovirus has an adjuvant effect. No ND vaccines based on the BEVS are available in the market. In the preclinical phase, the F or HN proteins are expressed in sf9 cells, and the antigens are harvested to prepare subunit vaccines [91, 92]. Due to the presence of major protective antigens, the immunogenicity and efficacy of subunit ND vaccines are comparable to those of whole-virus inactivated vaccines [92]. Notably, the extra antigen purification process and the resultant increase in costs are concerns about poultry subunit vaccines. A recent study showed that antigen purification may not be necessary for subunit vaccines. An H7N9 subtype AIV subunit vaccine produced in insect cells as a crude antigen is highly immunogenic and efficacious in chickens [93]. Considering the need to control costs, the production of subunit vaccines based on crude antigens expressed in the BEVS may be acceptable for poultry vaccines.

Plant-based expression systems are an attractive platform for the production of poultry subunit vaccines [94]. Protective F and/or HN antigens are expressed in various plants, such as *N. benthamiana*, *Zea mays*, *Solanum tuberosum* and *Oryza sativa* [94]. These antigens can elicit specific antibodies or cellular immunity in chickens or mice. When Shahid et al. expressed the HN protein in corn, and chickens orally fed leaves and seeds of maize mounted an NDV-specific antibody response [95]. A recombinant F protein produced in rice was shown to induce antibody immunity and provide complete protection against genotype VII NDV infection [96]. Moreover, an F-based subunit vaccine allows differentiation between infected and vaccinated animals (DIVA) by detecting HN-specific antibodies [96]. Therefore, plant-based systems are promising for the generation of novel ND vaccines due to advantages including low costs, no or little requirement for purification, a lack of pollution and oral administration.

### Virus-like particle vaccines

Another type of experimental ND vaccine is the virus-like particle (VLP) vaccine. VLPs resemble natural virus particles in terms of their morphology and size, while they contain no nucleic acids. VLP vaccines are safe in target animals because they cannot replicate in the host. Protective antigens that can stimulate cellular and antibody immunity are incorporated into VLPs. The M protein is the main driving force of NDV assembly and budding [97]. The expression of a single M protein is sufficient for VLP assembly, and thus, the M protein is obligatory for the production of NDV VLPs. The F, HN and M proteins are expressed in the BEVS for VLP assembly, and VLPs are purified from the samples to prepare vaccines [98, 99]. Xu et al. generated a VLP vaccine candidate against genotype VII NDV by coexpressing the M, F and HN genes in sf9 cells [99]. An alum-adjuvanted VLP vaccine was shown to induce a longer protection period and a shorter virus shedding period than the whole-virus inactivated La Sota vaccine when challenged with the genotype VII NDV strain [99]. In addition, NDV VLPs can also be used as a platform for generating vaccines for human and agricultural pathogens [100, 101]. Because VLP assembly is dependent on interactions among different viral proteins, experimental parameters must be optimized to maximize the yield of VLPs. Antigen purification steps are required for the preparation of VLP vaccines.

### Vaccines for differentiation between infected and vaccinated animals

The ultimate goal of animal infectious disease control is disease eradication. The DIVA strategy is critical for this purpose [102]. In countries where ND is endemic, DIVA vaccines are helpful for monitoring virus circulation in vaccinated flocks. In countries where ND is well controlled using vaccination programs, such as China, DIVA vaccines are of great importance for next-step disease elimination.

Since all current ND vaccines contain whole-virus components, antibodies induced by these vaccines are not readily distinguished from those induced by virus infection using traditional serological tests. Based on the aforementioned profiles of different ND vaccines, virus-vectored vaccines, subunit vaccines and VLP vaccines can be used as DIVA vaccines because they only contain F and/or HN proteins (whereas VLPs also contain the M protein). Therefore, DIVA can be performed by detecting antibodies against internal viral proteins such as the NP, P or L protein. For example, as described above, the F protein-based subunit vaccine produced in rice is highly protective, and DIVA can be performed by detecting HN-specific antibodies using immunochromatographic strips within 10 min [96]. In addition, enlightened by the low cross-reactivity between NDV and other APMVs, Peeters et al. generated a recombinant NDV harbouring a chimeric HN protein composed of the globular head of APMV-4 and the stalk region of NDV [103]. This virus is fully protective against virulent NDV. More importantly, using the APMV-4-specific HI assay and ELISA targeting the HN ectodomain, clear discrimination between the sera induced by the recombinant and La Sota vaccines was accomplished. A previous study reported an epitope-deletion strategy for generating an NDV vaccine carrying a DIVA marker [104]. An 18-amino acid immunodominant epitope (IDE) in the NP protein was replaced by a B-cell epitope of the S2 glycoprotein of murine hepatitis virus (MHV). ELISA against the IDE of NP and the MHV S2 epitope can achieve the goal of DIVA. All of the strategies described above present potential for the generation of DIVA ND vaccines. It is noteworthy that ideal DIVA vaccines should be highly efficacious and harbour dominant markers allowing specific, sensitive and effective detection.

### Immunomodulators of ND vaccines

Cytokines are critical components of the innate and adaptive immune responses and can thus be used as immunostimulatory adjuvants for vaccine preparation. Many studies have demonstrated that the codelivery of chicken cytokines, such as interleukin (IL)-1β, IL-12, IL-18 and granulocyte monocyte colony stimulating factor (GM-CSF), through different routes can significantly enhance the immunogenicity and efficacy of ND vaccines [105–107]. Here, we focus on discussing the strategy of cytokine expression in the NDV backbone as a foreign gene. Zhang et al. reported that recombinant NDVs expressing IL-2, IL-15 or GM-CSF elicited a quick antibody response in chickens (Day 8 post vaccination) and conferred complete protection against virulent NDV challenge [108]. In particular, the virus expressing GM-CSF appeared to be resistant to the influence of MDA. The authors speculated that the expression of cytokine adjuvants could potently induce the maturation of T cells to activate B-cell responses. In addition, an attenuated NDV expressing interferon (IFN)-γ has been assessed either by in ovo immunization as a live vaccine or by injection as an inactivated vaccine [109]. Unfortunately, regardless of the delivery route, IFN-γ failed to enhance the immunogenicity of the NDV vaccine. In addition, the virulent version of the IFN-γ-expressing NDV displayed decreased pathogenicity in 4-week-old chickens, as evidenced by a lack of mortality, decreased disease severity and virus shedding [110]. These findings suggest that IFN-γ may act as an antiviral factor and has no augmenting effect on virus immunogenicity. Interestingly, two independent studies revealed a robust function of an antisense IL-4 gene in regulating NDV immunogenicity [111, 112]. Collectively, the data from these two works indicated that in ovo immunization with attenuated NDVs carrying an antisense IL-4 gene induced an early antibody response and high protection in commercial chicken eggs with high MDA titres. More importantly, these vaccines significantly increased posthatch survival and body weight gain, highlighting their promise for serving as ideal in ovo ND vaccines. Because the antisense sequence of the IL-4 gene is inserted into the NDV genome, the IL-4 protein is not expressed during virus replication. Instead, the existence of the antisense IL-4 RNA produced by the virus during replication may be associated with the observed phenotypes of the recombinant NDV. However, the effects of antisense IL-4 RNA on the host immune response and virus immunogenicity are still unclear. Recently, Liu et al. revealed that the expression of the IFN-stimulated gene 12–2 leads to the attenuation and increased immunogenicity of the ND vaccine, which may be attributed to the regulatory activity of this gene toward innate and adaptive immune responses [113]. Therefore, the expression of cytokines or cytokine-related gene products in NDV as molecular adjuvants or regulators may be a promising pathway for enhancing the immunogenicity and capacity of ND vaccines to overcome MDA interference.

## Perspectives for disease control in different countries

Based on the specific disease situation and risk of disease occurrence, countries can be classified into three tiers, and different strategies for vaccination and future vaccine development are proposed accordingly.

Tier 1: ND-nonendemic countries. Most developed countries belong to this tier. Due to rigorous biosecurity and stamping-out policies, there are only sporadic occurrences of ND, and the risk of virus spreading in these countries is low. Therefore, routine vaccination programmes are effective for disease prevention and control in commercial flocks. To further increase the efficiency of ND vaccination, it is critical to develop novel ND vaccines suitable for in hatchery immunization, such as in ovo vaccines for broilers. In addition, the development of cell-based ND vaccines should be considered as an option because the egg supply is the bottleneck in the production of traditional vaccines, and poultry vaccines could be produced in a more environmentally friendly manner.

Tier 2: ND-endemic but well-controlled countries. China is a typical example of such a country. ND has been endemic in these countries in past decades, but the disease has been well contained recently because of the application of genotype-matched vaccines and the enforcement of biosecurity. It is vital to maintain current vaccination programmes and to develop novel DIVA vaccines, which are essential for monitoring virus dissemination in poultry and disease eradication in the long term. Moreover, to match the fast development of the poultry industry, the vaccination strategy should be revised, and next-generation ND vaccines allowing mass administration in hatcheries, such as through in ovo injection and spraying, are therefore in great demand.

Tier 3: ND-endemic countries. Most developing countries can be grouped in this tier. Due to poor biosecurity policies or the improper storage or administration of vaccines, poultry flocks are exposed to high levels of virulent viruses spreading in the field. Thus, the implementation of strict biosecurity measures, the development of novel vaccines matching the dominant viruses and adequate administration in the field are important, as these strategies can decrease the amount of virus shedding and circulation in the flocks. In addition, in some developing countries, due to the shortage of cold chains and well-trained veterinarians, thermotolerant ND vaccines that can be administered easily may be preferentially accepted by farmers.

## Conclusions

In contrast to the situation in some developed countries, ND continues to occur in most developing countries and is of great concern in the context of poultry-associated food security. The extensive application of currently available vaccines plays a significant role in disease prevention and control in developed and developing countries. Because NDV is highly contagious and wild birds can sometimes carry the virus without becoming ill, ND outbreaks can occur anywhere that poultry are raised. In addition, the disease situations in different countries and poultry farms are distinct, highlighting the fit-for-purpose application of ND vaccines and the development of next-generation ND vaccines.

## Data Availability

Data sharing is not applicable as no datasets were generated or analysed for this article.

## Abbreviations

AIV: avian influenza virus
APMV: avian paramyxovirus
BEVS: baculovirus expression vector system
DIVA: differentiation between infected and vaccinated animals
ECEs: embryonated chicken eggs
EID_50_: 50% embryo infectious dose
F: fusion
FPV: fowlpox virus
GM-CSF: granulocyte monocyte colony-stimulating factor
HA: haemagglutinin
HI: haemagglutination-inhibition
HN: haemagglutinin-neuraminidase
HVT: turkey herpesvirus
IBDV: infectious bursa disease virus
IBV: infectious bronchitis virus
ICPI: intracerebral pathogenicity index
IDE: immunodominant epitope
IFN: interferon
IL: interleukin
L: large protein
M: matrix
MDA: maternally derived antibody
MDT: mean death time of embryos
MDV: Marek’s disease virus
MHV: murine hepatitis virus
ND: Newcastle disease
NDV: Newcastle disease virus
NP: nucleoprotein
OIE: World Organisation for Animal Health
P: phosphoprotein
PPMV: pigeon paramyxovirus
s.c.: subcutaneous
VLP: virus-like particle
